# Characteristics of Stress and Suicidal Ideation in the Disclosure of Sexual Orientation among Young French LGB Adults

**DOI:** 10.3390/ijerph15020290

**Published:** 2018-02-07

**Authors:** Elodie Charbonnier, Florence Dumas, Adam Chesterman, Pierluigi Graziani

**Affiliations:** 1 LPS (Laboratoire Psychologie Sociale), Aix Marseille University, 13100 Aix en Provence, France; pierluigi.graziani@unimes.fr; 2 Department Psychology, Letter, Languages and History, LPS (Laboratoire Psychologie Sociale), 30000 Nimes, France; 3 Department Psychology, Letter, Languages and History, Laboratoire CHROME (Détection, Evaluation, Gestion de Risques CHOniques et éMergenst), 30000 Nimes, France; florence.dumas@unimes.fr (F.D.); adam.chesterman@unimes.fr (A.C.)

**Keywords:** suicide, stress, disclosure of sexual orientation, LGB young adults

## Abstract

*Background*: Lesbians, gays, and bisexual people (LGB) present high levels of suicidal ideation. The disclosure of sexual orientation is a stressful experience which presents a high suicide risk. Research has not paid sufficient attention to stress during this disclosure in order to understand suicide among LGB people. The aims of this study were to investigate: (1) the characteristics of stress during this revelation, more precisely cognitive appraisal, emotions, and coping; and (2) associations between these characteristics and suicidal ideation. *Method*: A total of 200 LGB young adults answered the “Stressful situation assessment questionnaire”, focusing on the most stressful disclosure of sexual orientation they have ever experienced. *Results*: Avoidance coping is a good predictor of suicidal ideation, and mediates the association between primary appraisal (risk “Harm myself and others”) and suicidal ideation. *Conclusions*: Our study illustrates the need to better understand stress during the disclosure of sexual orientation to prevent and care for suicide risk among LGB young adults.

## 1. Introduction

Lesbians, gays, and bisexual people (LGB) present higher levels of self-harm, suicidal ideation, and attempted suicide than heterosexuals [1,2,3,4,5]. French studies have yielded similar results [6,7,8,9]. Suicide attempts are nearly 2.5 times more likely to occur in LGB youths rather than heterosexual youths [10]. LGB people are exposed to high levels of stress, and the multiplication of stressful experiences such as prejudice, rejection, or discrimination [11] can lead to suicidal ideation [12]. In light of this, this study investigated the role of stress as one underlying factor that is likely to contribute to suicidal ideation. The “minority stress model” (MSM) [13,14,15] is particularly relevant for understanding high stress levels in LGB people. This model postulates that people from stigmatized social categories (such as LGB people) are more exposed to stress because they are exposed to specific stressors described as “minority stress” [16]. LGB people have four major “minority stresses”: discrimination experiences, concealment or disclosure of sexual orientation, expectations of prejudice and discrimination, and internalized homonegativity [13,17].

The present study focused specifically on one minority stress, the disclosure of sexual orientation, because it is an essential component in LGB identity formation and integration [18,19], and is associated with a high risk of suicide [20,21,22]. For LGB people, deciding to disclose their sexual orientation involves examining feelings that may inhibit the will to disclose, and gaining greater clarity about the personal meaning of being gay, lesbian, or bisexual. It is an emotional and cognitive process that begins before disclosure and endures well after, when the person continues to reflect on his/her sexual orientation and revelations that are still to come [23]. The disclosure of sexual orientation is related to various benefits (e.g., well-being, reduced stress, better relationships) [19,24,25,26]. However, this disclosure can also have deleterious effects on physical and psychological health [27], on discrimination and prejudice [13,27,28], and peer victimization and harassment [29]. Indeed, disclosing one’s sexual orientation implies defining oneself as different, outside the norm [30], and thus facing possible judgment and rejection [13,31]. The disclosure of sexual orientation is associated with a high suicide risk [20,22,28], which is increased when disclosure leads to family rejection [32,33]. 

In sum, there is clear evidence that the disclosure of sexual orientation is a stressful experience with a high risk of suicide. Surprisingly, research has generally ignored the role of characteristics of stress such as emotions, cognitions, and coping during the disclosure of sexual orientation. The “transactional model of stress” highlights the critical importance of taking these characteristics into account [34,35,36]. This model suggests that stress is the result of a cognitive and emotional appraisal that leads to coping. Cognitive appraisal of a stressful situation is characterized by two cognitive mechanisms. During the primary appraisal, the person evaluates what is at stake in the situation. During the secondary appraisal, he or she assesses his/her resources and alternatives to manage the situation (perceived control). The less an individual perceives control during a stressful encounter, the more he/she feels negative emotions. Thus, emotions depend on cognitive appraisals and motivated coping strategies [37]. 

Some scholars highlight the critical importance of emotions (especially negative emotional states) [38,39,40,41], cognitive appraisal [42], and coping [38,43,44], on suicide. To date, however, these relationships have not been studied among LGB people, although Hatzenbuehler (2009) [45] showed that coping mediates the link between minority stress and mental health. Consequently, the present study combined the minority stress model [13] and the transactional model of stress [34,35,36] in an effort to investigate: (1) stress (specifically, cognitive appraisal, emotions, and coping) during the disclosure of sexual orientation among young French LGB adults; and (2) associations between characteristics of stress and suicidal ideation during this disclosure. 

## 2. Methods 

### 2.1. Participants

A total of 200 self-identified LGB French young adults responded, including 69 women and 131 men, 62 who were lesbian, 124 who were gay, and 14 who were bisexual, ranging from 18 to 28 years of age (m = 21.7, SD = 2.6). Participants were French citizens from various French regions. In the sample, 55% lived in town centers (*n* = 110), 33.5% in rural areas (*n* = 67), 8.5% in middle class suburbs (*n* = 17), and 3% in disadvantaged suburbs (*n* = 6). Regarding conjugal status, 74.5% were single (*n* = 149), 24% lived with a partner (couple, married or civil partnership) (*n* = 48), and three did not respond. The mean age for their most stressful sexual orientation disclosure was 18.2 years (SD = 2.57; minimum = 12; maximum = 26), on average 3.5 years ago (SD = 2.39; minimum = 0; maximum = 12), and they have lived on average 2.4 stressful disclosures of their sexual orientation over the course of their lives (SD = 1.70; minimum = 0; maximum = 5). Individuals involved in these stressful disclosures were parents (*n* = 131; 65.50%), friends (*n* = 28; 14%), other family members (*n* = 25; 12.50%), family and friends (*n* = 9; 4.50%), work (*n* = 3; 1.50%), and other (*n* = 4; 2%). 

### 2.2. Procedure 

Data for this study were collected from two French LGB dating sites (Gaypax: www.gaypax.com; Gayvox: www.gayvox.fr). Private emails were sent to all profiles describing the theme, framework, and procedure. To those interested, we sent a confidential questionnaire with an explanation of the research. Once they had provided their informed consent, interested individuals received an anonymous questionnaire online. They signed a consent form and were informed that their information would remain anonymous and their participation was voluntary and could be withdrawn at any time. All data were collected online and were stored on a secure university computer and removed from our inboxes. Later, we contacted the individuals again (by email or telephone) to answer any questions they may have had. This research was conducted during a PhD study in France under the supervision of a Full Professor. We respected the rules of the Declaration of Helsinki of 1975. 

### 2.3. Measures

Participants had the following instructions: “Consider your most stressful disclosure of your sexual orientation and answer the following questions”. Emotions, cognitive appraisal, coping, and suicidal ideations during this disclosure were evaluated. Most tools assessing the characteristics of stress focus on a recent stressful event (during the last week, the last month) and/or invite participants to choose a situation. To achieve our objectives, we imposed a specific stressful situation (i.e., the most stressful disclosure of sexual orientation), without time constraints. In addition, no measures of stress have been specifically validated among LGB people. Therefore, in our protocol, we used the “stressful situation evaluation questionnaire” (Le Questionnaire d’Evaluation de la Situation Stressante—QETS) [46,47], which assesses different dimensions of stress according to the transactional model (emotions, cognitive appraisals, and coping). In our study, we replaced the original QETS instruction (i.e., “a recent stressful situation in your daily life”) with “the most stressful disclosure of your sexual orientation in your life”. 

#### 2.3.1. Emotions

As in the QETS, we evaluated the intensity of 23 emotions on five-point Likert scales (0 = “not at all” to 4 = “very”). Participants evaluated emotions both at the beginning and at the end of their most stressful disclosure. In the QETS, authors used four emotional factors, but in our sample, the internal consistency of these factors (at the beginning and at the end of the disclosure) was unreliable. As such, emotional factors were identified using principal component analysis with varimax rotation, at the beginning and at the end of the disclosure situation. Items with factor loadings of 0.30 or higher were considered as being strongly related to the corresponding factor. 

We identified five factors for the beginning of the disclosure situation, which explained 57.94% of the overall variance:

− “Well-being” (23.07% of explained variance) included six emotions (amused, reassured, satisfied, happy, hopeful, relieved).
− “Hostility and disappointment” (14.96% of explained variance) included six emotions (angry, sad, disappointed, offended, disgusted, frustrated).
− “Anxious anticipation” (9.91% of explained variance) included six emotions (preoccupied, threatened, scared, guilty, anxious, embarrassed).
− “Mastery” (5.38% of explained variance) included three emotions (combative, confident, master of myself).
− “Jealousy” (4.66% of explained variance) included two emotions (jealous, envious).

At the end of the disclosure situation, we identified four factors that accounted for 63.05% of the explained variance:

− “Well-being and mastery (At the end, two initials factors (well-being and mastery) are grouped as a single factor)” (38.57% of explained variance) included nine emotions (amused, reassured, satisfied, happy, hopeful, relieved, combative, confident, master of myself).
− “Hostility and disappointment” (13.46% of variation explained) included six emotions (angry, sad, disappointed, offended, disgusted, frustrated).
− “Anxious anticipation” (5.88% of variation explained) included six emotions (preoccupied, threatened, scared, guilty, anxious, embarrassed).
− “Jealousy” (5.14% of variation explained) included two emotions (jealous, envious).

#### 2.3.2. Cognitive Appraisals 

As in the QETS, for the primary appraisals (assessment of what is at stake, risk, and threat), participants rated the importance of 13 general risks (e.g., hurt a relative, lose your self-esteem, appear as an unethical person, diminish your financial resources, etc.). In the original version, four risk factors were retained, but in our sample, the internal consistency of these factors was unreliable. Because of this, as for emotions, risk factors were identified using principal component analysis with varimax rotation and we retained items with factor loadings of 0.30. We identified five factors that account for 65.16% of the overall variance:

− Risk 1 “Loss of respect and affection” (25.49% of explained variance) included three items: “Lose the affection of someone who is important to you”; “Lose the approval or respect of someone who is important to you”; “Lose respect for someone else”.
− Risk 2 “Appear as a careless, unethical person” (12.94% of explained variance) included two items: “Appear as a careless person”; “Appear as an unethical person”.
− Risk 3 “Incompetence and failure” (9.94% of explained variance) included three items: “To not reach an important goal at work”; “Diminish your financial resources”; “Come across as incompetent”.
− Risk 4 “Cause suffering” (8.86% of explained variance) included two items: “Harm the physical wellbeing and health of your relatives”; “Hurt a relative”.
− Risk 5 “Harm myself or others” (7.94% of explained variance) included three items: “Jeopardize your own physical health”, your security or your wellbeing”; “Disrupt a relative’s habits”; “Lose your self-esteem”.

For the secondary appraisal (perceived control), as in the QEST, participants evaluated four coping strategies: “Change the situation or act upon it”, “Accept the situation”, “Seek information before acting”, or “Stick to the plan and keep doing what you wanted to do”. They answered on five-point Likert scales (0 = “not at all” to 4 = “a lot”).

#### 2.3.3. Coping 

Coping was evaluated with the revised French version of the Ways of Coping Check-List [46]. It contains 67 items with a wide range of thoughts and acts that people use to deal with the internal and/or external demands of specific stressful encounters. Respondents answered on four-point Likert scales (0 = “not at all”; 3 = “a lot”). The French version includes 10 factors, but in our sample, the internal consistency of these factors was unreliable. As such, we again chose to conduct principal component analysis (with varimax rotation). We identified 22 factors and we retained eight. The percentage of explained variance was 40.52%:

− “Avoidance” (12.05% of explained variance) included twenty two items (items 3, 6, 9, 11, 12, 14, 21, 25, 28, 32, 33, 34, 35, 41, 44, 51, 56, 57, 58, 59, 60, 62).
− “Positive development” (9.01% of explained variance) included ten items (items 15, 18, 20, 23, 29, 30, 37, 39, 47, 50).
− “Seeking help” (4.09% of explained variance) included six items (items 8, 18, 31, 43, 46, 53).
− “Preparation and escape” (3.64% of explained variance) included ten items (items 19, 26, 27, 36, 52, 58, 60, 63, 54, 67).
− “Minimization” (3.21% of explained variance) included six items (items 13, 24, 54, 42, 45, 55).
− “Problem solving” (2.99% of explained variance) included eight items (items 1, 2, 5, 10, 22, 61, 65, 66).
− “Aggression” (2.95% of explained variance) included three items (items 17, 40, 48).
− “Acceptance” (2.57% of explained variance) included five items (items 4, 7, 20, 34, 38).

#### 2.3.4. Suicidal Ideation 

Suicidal ideation following the disclosure of sexual orientation was assessed with one item (“I thought of killing myself for a brief moment”) on a four-point Likert scale, from 0 (“not at all”) to 3 (“a lot”).

### 2.4. Data Analysis

Data were analyzed using SPSS version 23 (IBM Inc., Chicago, IL, USA). Descriptive statistics were used to investigate the components of stress during the disclosure of sexual orientation: cognitive appraisal, emotions, and coping strategies. Afterwards, two sets of stepwise regression analyses were conducted on suicidal ideation. The first set was used to examine whether coping strategies and emotional appraisals at the end of the disclosure of sexual orientation were significant predictors of suicidal ideation. The second set of stepwise regression analyses aimed to test whether adding the primary and secondary cognitive appraisals or emotions before the disclosure increased the comprehensive model of suicidal ideation. In other words, this approach allowed us to determine whether cognitive appraisals and emotions explain a substantial proportion of suicidal ideation variance. 

## 3. Results

The components of stress related to participants’ most stressful sexual orientation disclosure are reported in Table 1 (mean and standard deviations). At the moment of their disclosure, 38% (*n* = 76) have at least thought “a little” about killing themselves. For primary appraisal, the most important risks are loss of respect, loss of someone’s affection, and to either suffer themselves or cause suffering in others. The main resource they think they have is acceptance. They consider that they cannot change or act upon their disclosure. Emotions described at the start of the disclosure situation refer most to anxiety. After disclosure, we observed increased well-being and mastery, and decreased anxiety. To deal with disclosing their sexual orientation, respondents call upon quite varied coping strategies, among which seven factors of coping had very close means. Participants essentially used positive development strategies, and hence tried to see the good side of the situation and help seeking. That said, they also called upon problem solving coping strategies, as well as acceptance, minimization and avoidance. For all variables, we observed no effects of age of disclosure, or sexual orientation, except for three dimensions which were higher for gays: anxiety in the beginning (m_Gays_ = 2.49; SD_Gays_ = 0.84; m_Lesbians_ = 2.42; SD_Lesbians_ = 0.91; m_Bisexuals_ = 1.74; SD_Bisexuals_ = 0.90; F = 4.72; *p* = 0.01); risk “incompetence and failure” (m_Gays_ = 0.61; SD_Gays_ = 0.83; m_Lesbians_ = 0.37; SD_Lesbians_ = 0.56; m_Bisexuals_ = 0.12; SD_Bisexuals_ = 0.30; F = 4.35; *p* = 0.01); and well-being at the end (m_Gays_ = 2.02; SD_Gays_ = 1.07; m_Lesbians_ = 1.78; SD _Lesbians_ = 1.12; m_Bisexuals_ = 1.21; SD_Bisexuals_ = 0.95; F = 3.94; *p* = 0.02). 

The first set of stepwise regression analyses, conducted on suicidal ideation, with coping strategies and emotional appraisals as predictors, showed that only avoidance coping and positive development coping were significant predictors. Avoidance coping was found to be the better predictor and to be positively associated with suicidal ideation (B = 0.58). This positive association can be described as follows: the stronger the avoidance strategy, the stronger the suicidal ideation score. A negative association was obtained for positive development coping and suicidal ideation (B = −0.16). This negative association can be described as follows: the stronger the positive development strategy, the weaker the suicidal ideation score. The three other coping strategies, as well as the five emotional factors at the end of the disclosure situation, were not significant predictors. They were excluded from the best model (Model 1) reported in Table 2, which explained 36% of the total variance. When avoidance coping was the only predictor in the equation, the percentage of explained variance was already 33%, thus highlighting the crucial role of avoidance coping in suicidal ideation. In the second set of stepwise regression analyses, primary and secondary cognitive appraisals, as well as emotions before disclosure, were added to Model 1. None of the emotions or secondary cognitive appraisals were statistically significant predictors of suicidal ideation. Only two of the four primary cognitive appraisals were significant predictors. Coefficients were positive, ranging from 0.15 to 0.13 for Risk 3 (“Incompetence and Failure”) and for Risk 4 (“To cause suffering”), respectively. The percentage of explained variance for the best model (Model 2, Table 2) was 38%, which represents only a slight increase compared to Model 1. 

**Figure 1:**
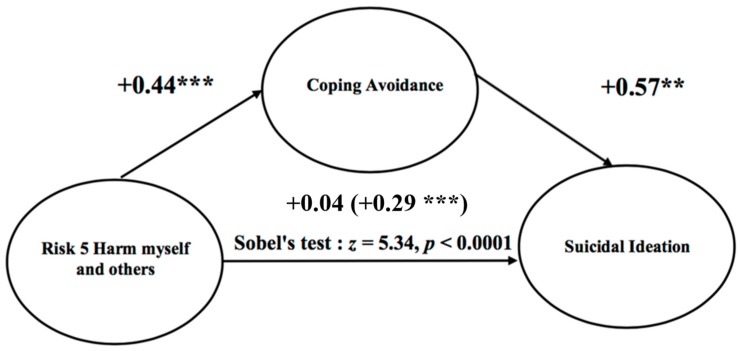
The effect of Risk 5 Harm myself and others for Suicidal Ideation after controlling for Coping Avoidance. The number in parentheses indicates the direct effect of Risk 5 on Suicidal Ideation prior to controlling for Coping Avoidance. ** *p* < 0.001. *** *p* < 0.001.

Complementary analyses revealed that avoidance coping completely mediates the effect of Risk 5 (“Harm myself and others”) on suicidal ideation (Figure 1). This mediation means that Risk 5 cannot be identified as a significant and independent predictor of suicidal ideation in our previous analyses. Our mediation test was conducted by following the four steps recommended by Baron and Kenny (1986) [48]. The direct effect of Risk 5 on suicidal ideation ratings was no longer significant after controlling for avoidance coping (*p* = 0.51), indicating that avoidance coping fully explains the effect of Risk 5 on suicidal ideation. The regression coefficient associated with the effect of Risk 5 on suicidal ideation varies from 0.29 to 0.04 when avoidance coping is controlled in the equation. This mediation still emphasizes the crucial role of avoidance coping in suicidal ideations. No other mediation effect was observed.

We examined whether the absence of an effect of the secondary cognitive appraisal was explained by an intercorrelation with primary cognitive appraisals. Of the six significant correlations, the two highest coefficients (*r* = 0.34 for “stick to the plan and keep doing what you wanted to do” and *r* = 0.22 for “seek information before acting”) were observed for Risk 3 (incompetence and failure). As Risk 3 had a significant effect on suicidal ideation, it is possible that the absence of an effect of secondary cognitive appraisals is at least partially due to these intercorrelations.

## 4. Discussion

Our study combined the minority stress model [13] and the transactional model [34,35,36] in an effort: (1) to understand the characteristics of a specific minority stress: the disclosure of one’s sexual orientation; and (2) to subsequently determine the effects of these characteristics on suicidal ideations. 

Concerning primary appraisal, LGB young adults are mostly fearful of losing a close one’s respect and affection, and making their relatives suffer. Therefore, their fears focus on the consequences of their disclosure on their relationships [49,50]. LGB people can be a burden for their relatives [51,52]. Frequently, before disclosure, they anticipate stigmatization, negative judgments, or feelings of rejection [53], and they fear losing friends and/or family [54]. Consequently, they remain concealed when they think they lack the necessary emotional resources to cope with others’ disapproval and anger [55,56]. They are also afraid that this disclosure could be harmful to them; they fear being hurt and jeopardizing their own physical health. The level of disclosure is guided by fears of prejudicial treatment and rejection [54,57]. Indeed, disclosing their sexual orientation can leave LGB people vulnerable to harassment, assault, or rejection [21]. As such, during their disclosure, LGB young adults fear rejection, loss of respect, suffering, and causing others to suffer, even though the main motivations and expectations of disclosure are to be accepted and supported [49,58]. Concerning secondary appraisal, the main resource participants think they have for their disclosure is acceptance. There is clear evidence that the disclosure of one’s sexual orientation is a process of acceptance and identity building [19], but which also implies accepting others’ reactions.

As for emotions, anxiety is dominant at the beginning of disclosure and can be linked to fears of being rejected, suffering, and causing suffering in others. At the end of the disclosure process, anxiety decreases while well-being and mastery increase. The act of using language (e.g., disclosing sexual orientation) provides the opportunity to think about and integrate stressful information in new and meaningful ways that can contribute to well-being [59,60]. Moreover, the disclosure of one’s sexual orientation also provides the opportunity for emotional processing that, in turn, may enable individuals to clarify and integrate their thoughts and feelings and thus promote the development of a stable, positive, and authentic identity [53,61]. 

Concerning coping, LGB young adults essentially use positive development strategies by focusing on the positive aspects of their disclosure. These results can be linked to stress-related growth theory, which postulates that personal growth is encouraged by stressful experiences [62,63], and has highlighted the relevance of considering the disclosure of one’s sexual orientation as a stress-related growth. Our results on coping strategies and emotions confirm that the disclosure of one’s sexual orientation is a process that encourages personal growth [64] and is an essential component in LGB identity formation and integration [18,19].

Secondly, we explored associations between characteristics of stress (emotions, cognitive appraisal, and coping) and suicidal ideation. Two risks were directly associated with suicidal ideation: “Incompetence and failure” and “To cause suffering”. Individuals decided to disclose their sexual orientation while also thinking that it would make people suffer, that they would come across as incompetent, that it would prevent them reaching goals and diminish financial resources, and that it could develop negative views of themselves and of their future. This hypothesis can be linked to Beck’s cognitive triad [42], which highlighted the role of negative views of the self and of the future in depression, and to major depressive disorder, which is strongly associated with suicidal ideations [65,66,67]. Views about oneself and the future have been very sparsely studied in the case of the disclosure of one’s sexual orientation, and developing these dimensions in the future for understanding suicide risk among LGB young adults during this disclosure is promising. Moreover, avoidance coping was the better predictor of high suicidal ideation. Furthermore, the fear of being hurt and of harming relatives during the disclosure of one’s sexual orientation increases avoidance coping, which in turn increases suicidal ideation. The role of avoidance coping is consistent with suicide literature [68,69,70,71], but our study is the first to confirm these associations for LGB young adults when disclosing their sexual orientation. Future research should continue investigating the role of cognitive appraisal and coping in the disclosure of sexual orientation, and also in other minority stress situations, in order to understand suicidal ideation among LGB people. These data confirm the therapeutic benefits of helping LGB young adults in their decision-making, by accompanying them in the cognitive appraisal of and coping with their revelation in order to prevent suicide. Considering the advantages and disadvantages of disclosure, evaluating the risks and exploring coping strategies for dealing with relatives’ reactions are all essential for deciding if and when to disclose. Thus, clients may benefit if therapists help them build the skills needed to cope with this revelation and its consequences, while also understanding the specifics of this minority stress. 

A number of methodological limits should be acknowledged. Firstly, our sample was mostly comprised of lesbian and gay people, and bisexual men and women were under-represented. The small sample size does not allow detailed comparisons between lesbian/gay individuals and bisexual men and women. For future studies, it would be interesting to explore stress in bisexual people when they reveal their bisexuality, especially since Fox [72] showed that the development of identity in bisexual people, which includes revealing one’s sexual orientation, can progress more slowly than in individuals who identify as gay or lesbian. Secondly, online recruitment is more likely to contain potential lies and errors. Yet, it is an efficient approach to recruitment in a specific, difficult-to-access population like LGB people [73]. Some important French research organisations (e.g., “Institut National de la Santé et de la Recherche Médicale” and “Institut de Veille Sanitaire”) have resorted to online recruiting [74]. Furthermore, our sample is composed solely of dating site users, which may have led to a lack of diversity in our population. Some authors have highlighted Internet use as a means to express and overcome difficulties [75,76]. Other authors have suggested that online communication can reduce symptoms of depression [77]. In light of this, our recruitment method can be criticized for its representativeness. That said, it was chosen in order to form a larger sample. In fact, studies have shown that a large proportion of LGB individuals now use the Internet to find partners, in particular among men, for whom proportions of 79.8% [78] and 97.0% [79] have been reported. In any case, our results can only be generalized to LGB people who use dating sites. Thus, the retrospective nature of our questionnaire (i.e., they responded on average 3.5 years after their most stressful disclosure) requires examining the results with much attention. As time goes by, events can be reinterpreted. In this way, the effect of time should lead to caution in the examination of our results. This limitation could reduce the reliability of emotional assessments and could explain the lack of effect of emotions on suicidal ideation. Moreover, LGB young adults who have recently disclosed their sexual orientation have experienced different stigma-related stresses than those who have lived with this disclosure for several years. Our participants had to answer our questions in reference to their most stressful disclosure experience, but some of them had probably experienced other disclosures since the most stressful one, which could lead them to restructure their perception. Disclosing one’s sexual orientation is a dynamic process in that its psychological consequences and impacts on identity change from one disclosure to the next. Furthermore, close-ones’ perception also changes over time and impacts identity and the perception of disclosure [50,55,80]. As such, isolating a specific moment in this process is complex, even though we made this choice in order to specifically understand the characteristics of stress in the disclosure of sexual orientation. Moreover, our results cannot be generalized to all disclosure situations, but only those that are highly stressful. 

## 5. Conclusions

Combining the minority stress model and the transactional model has significant potential for better understanding stress and suicide among LGB young adults. Our results highlighted characteristics of stress (fear, perceived control, coping, emotions) among LGB young adults during the disclosure of their sexual orientation, and links between these characteristics and suicidal ideations. More precisely, our study demonstrated that avoidance strategies had a direct effect on suicidal ideation, and mediated the link between primary appraisal (risk “Harm myself and others”) and suicidal ideation. Thus, our study illustrates the need to better understand stress during the disclosure of sexual orientation to prevent and care for suicide risk among LGB young adults. 

## Figures and Tables

**Table 1:** Means and Standard deviation of key variables (cognitive appraisal, emotions, and coping during the disclosure of sexual orientation the most stressful).

Variables	Mean (±SD)
**Primary appraisal**	
Risk “Lose respect and affection”	2.35 (±1.11)
Risk “Cause suffering”	1.92 (±1.21)
Risk “Harm myself or others”	1.54 (±0.96)
Risk “Appear as a careless, unethical person”	0.94 (±1.12)
Risk “Incompetence and failure”	0.5 (±0.74)
**Secondary appraisal**	
“Accept the situation”	2.98 (±1.25)
“Seek information before acting”	1.25 (±1.33)
“Stick to the plan and keep doing what you wanted to do”	1.23 (±1.45)
“Change the situation or act upon it”	0.89 (± 1.21)
**Emotional Factors at the beginning of the disclosure**	
Anxious anticipation	2.42 (±0.88)
Mastery	1.23 (±1.06)
Hostility and disappointment	1.21 (±1.08)
Well-being	1.08 (±0.96)
Jealousy	0.18 (±0.50)
**Emotional Factors at the end of the disclosure**	
Well-being and mastery	1.90 (±1.10)
Anxious anticipation	1.14 (±1.07)
Hostility and disappointment	1.06 (±1.16)
Jealousy	0.22 (±0.56)
**Coping strategies mobilised during disclosure**	
Positive development	1.51 (±0.66)
Help seeking	1.46 (±0.73)
Minimization	1.25 (±0.59)
Problem solving	1.21 (±0.48)
Acceptance	1.14 (±0.53)
Avoidance	1.10 (±0.57)
Preparation and escape	0.98 (±0.61)
Aggression	0.59 (±0.66)

**Table 2:** Results of the two sets of stepwise regression analyses on suicidal ideation.

Model	Standardized B	SE	*t*	df	Adjusted *R*^2^
**Model 1**				195	0.36
Avoidance coping	0.58 ***	0.11	10.18		
Development coping	−0.16 **	0.10	−2.75		
**Model 2**				194	0.38
Avoidance coping	0.50 ***	0.12	8.21		
Development coping	−0.17 **	0.10	−3.09		
Risk 3 Incompetence and Failure	0.15 *	0.10	2.44		
Risk 4 To cause suffering	0.13 *	0.05	2.16		

Note: * *p* < 0.05, ** *p* < 0.01, *** *p* < 0.001. Adjusted *R*^2^ values are significant at 0.001. Only the best model for each set of analyses is presented. Excluded variables are not reported.

## References

[1] Almazan E.P., Roettger M.E., Acosta P.S. (2014). Measures of Sexual Minority Status and Suicide Risk among Young Adults in the United States. Arch. Suicide Res..

[2] Johnson R.B., Oxendine S., Taub D.J., Robertson J. (2013). Suicide Prevention for LGBT Students. New Dir. Stud. Serv..

[3] Marshal M.P., Dietz L.J., Friedman M.S., Stall R., Smith H.A., McGinley J., Thoma B.C., Murray P.J., D’Augelli A.R., Brent D.A. (2011). Suicidality and Depression Disparities Between Sexual Minority and Heterosexual Youth: A Meta-Analytic Review. J. Adolesc. Health.

[4] Russell S., Joyner K. (2001). Adolescent sexual orientation and suicide risk: Evidence from a national study. Am. J. Public Health.

[5] Skerrett D., Kõlves K., Leo D. (2015). De Are LGBT populations at a higher risk for suicidal behaviors in Australia? Research findings and implications. J. Homosex..

[6] Bourdet S., Pugnière J.M., Welzer-Lang D. (2011). Attirance sexuelle, suicidalité et homophobie intériorisée. Masculinité état des Lieux.

[7] Jouvin E., Beaulieu-Prévost D., Julien D., Beck F., Guilbert P. (2007). Minorités sexuelles: Des populations plus exposées que les autres?. Baromètre Santé 2005.

[8] Velter A. (2007). Rapport Enquête Presse Gay 2004.

[9] Paget L.M., Chee C.C., Sauvage C., Saboni L., Beltzer N., Velter A. (2016). Factors associated with suicide attempts by sexual minorities: Results from the 2011 gay and lesbian survey. Rev. Epidemiol. Sante Publique.

[10] King M., Semlyen J., Tai S.S., Killaspy H., Osborn D., Popelyuk D., Nazareth I. (2008). A systematic review of mental disorder, suicide, and deliberate self harm in lesbian, gay and bisexual people. BMC Psychiatry.

[11] Lewis R.J., Derlega V.J., Griffin J.L., Krowinski A.C. (2003). Stressors for Gay Men and Lesbians: Life Stress, Gay-Related Stress, Stigma Consciousness, and Depressive Symptoms. J. Soc. Clin. Psychol..

[12] Almeida J., Johnson R.M., Corliss H.L., Molnar B.E., Azrael D. (2009). Emotional distress among LGBT touth: The influence of perceived discrimination based on sexual orientation. J. Youth Adolesc..

[13] Meyer I. (2003). Prejudice, social stress, and mental health in lesbian, gay, and bisexual populations: Conceptual issues and research evidence. Psychol. Bull..

[14] Meyer I., Dietrich J., Schwartz S. (2008). Lifetime prevalence of mental disorders and suicide attempts in diverse lesbian, gay, and bisexual populations. Am. J. Public Health.

[15] Meyer I.H., Northridge M.E. (2007). The Health of Sexual Minorities: Public Health Perspectives of Lesbian, Gay, Bisexual and Transgender Populations.

[16] Meyer I.H. (1995). Minority stress and mental health in gay men. J. Health Soc. Behav..

[17] DiPlacido J., Herek G.M. (1998). Minority stress among lesbians, gay men and bisexuals: A consequence of heterosexism, homophobia and stigmatization. Stigma and Sexual Orientation.

[18] Cass V.C. (1979). Homosexual identity formation: A theoretical model. J. Homosex..

[19] Rosario M., Hunter J., Maguen S., Gwadz M., Smith R. (2001). The Coming-Out Process and Its Adaptational and Health-Related Associations among Gay, Lesbian, and Bisexual Youths: Stipulation and Exploration of a Model. Am. J. Community Psychol..

[20] D’Augelli A., Grossman A., Salter N., Vasey J., Starks M., Sinclair K. (2005). Predicting the suicide attempts of lesbian, gay, and bisexual youth. Suicide Life Threat. Behav..

[21] D’Augelli A., Grossman A., Starks M. (2006). Childhood gender atypicality, victimization, and PTSD among lesbian, gay, and bisexual youth. J. Interpers. Violence.

[22] Dragowski E.A., Halkitis P.N., Grossman A.H., D’Augelli A.R. (2011). Sexual Orientation Victimization and Posttraumatic Stress Symptoms among Lesbian, Gay, and Bisexual Youth. J. Gay Lesbian Soc. Serv..

[23] Beals K.P., Peplau L.A., Gable S.L. (2009). Stigma management and well-being: The role of perceived social support, emotional processing, and suppression. Pers. Soc. Psychol. Bull..

[24] Critcher C., Ferguson M. (2014). The cost of keeping it hidden: Decomposing concealment reveals what makes it depleting. Exp. Psychol. Gen..

[25] Griffith K.H., Hebl M.R. (2002). The disclosure dilemma for gay men and lesbians: “Coming out” at work. J. Appl. Psychol..

[26] LaSala M. (2000). Gay male couples: The importance of coming out and being out to parents. J. Homosex..

[27] Frost D.M., Lehavot K., Meyer I.H. (2015). Minority stress and physical health among sexual minority individuals. J. Behav. Med..

[28] Pilkington N.W., D’Augelli A.R. (1995). Victimization of lesbian, gay, and bisexual youth in community settings. J. Community Psychol..

[29] Guzzo G., Pace U., Lo Cascio V., Craparo G., Schimmenti A. (2014). Bullying Victimization, Post-Traumatic Symptoms, and the Mediating Role of Alexithymia. Child Indic. Res..

[30] Bonet L., Wells B.E., Parsons J.T. (2007). A positive look at a difficult time: A strength based examination of coming out for lesbian and bisexual women. J. LGBT Health Res..

[31] D’Augelli A.R. (2002). Mental Health Problems among Lesbian, Gay, and Bisexual Youths Ages 14 to 21. Clin. Child Psychol. Psychiatry.

[32] Ryan C., Huebner D., Diaz R.M., Sanchez J. (2009). Family rejection as a predictor of negative health outcomes in white and Latino lesbian, gay, and bisexual young adults. Pediatrics.

[33] McConnell E.A., Birkett M., Mustanski B. (2016). Families Matter: Social Support and Mental Health Trajectories among Lesbian, Gay, Bisexual, and Transgender Youth. J. Adolesc. Health.

[34] Coyne J.C., Lazarus R.S., Kutasha I.L. (1980). Cognitive style, stress perception and coping. Hand Book of Stress and Anxiety: Knowledge, Theory and Treatment.

[35] Lazarus R.S. (1966). Psychological Stress and Coping Process.

[36] Lazarus R.S., Folkman S. (1984). Stress, Appraisal and Coping.

[37] Folkman S., Lazarus R. (1980). An analysis of coping in a middle-aged community sample. J. Health Soc. Behav..

[38] Baumeister R.F. (1990). Suicide as escape from self. Psychol. Rev..

[39] Lester D. (1997). The role of shame in suicide. Suicide Life Threat. Behav..

[40] Mokros H.B. (1995). Suicide and Shame. Am. Behav. Sci..

[41] Everall R.D. (2006). Being in the Safety Zone: Emotional Experiences of Suicidal Adolescents and Emerging Adults. J. Adolesc. Res..

[42] Beck A.T. (1979). 1921-Cognitive Therapy of Depression.

[43] Linehan M. (1993). Cognitive-Behavioral Treatment of Borderline Personality Disorder.

[44] Nock M.K., Wedig M.M., Holmberg E.B., Hooley J.M. (2008). The emotion reactivity scale: Development, evaluation, and relation to self-injurious thoughts and behaviors. Behav. Ther..

[45] Hatzenbuehler M.L., Dovidio J.F., Nolen-Hoeksema S., Phills C.E. (2009). An implicit measure of anti-gay attitudes: Prospective associations with emotion regulation strategies and psychological distress. J. Exp. Soc. Psychol..

[46] Graziani P., Rusinek S., Servant D., Hautekeete-Sence D., Hautekeete M. (1998). Validation française du questionnaire de coping “way of coping check-list-revised” (W.C.C.-R.) et analyse des événements stressants du quotidien. J. Ther. Comport. Cognit..

[47] Graziani P. (2001). Spécificité de L’évaluation, du Vécu Émotionnel et du Coping des Sujets Souffrant de Troubles Anxieux Confrontés à des Évènements Stressants. Ph.D. Thesis.

[48] Baron R.M., Kenny D.A. (1986). The moderator–mediator variable distinction in social psychological research: Conceptual, strategic, and statistical considerations. J. Personal. Soc. Psychol..

[49] Corrigan P.W., Larson J.E., Hautamaki J., Matthews A., Kuwabara S., Rafacz J., Walton J., Wassel A., O’Shaughnessy J. (2009). What Lessons do Coming Out as Gay Men or Lesbians have for People Stigmatized by Mental Illness?. Community Ment. Health J..

[50] Savin-Williams R.C., Ream G.L. (2003). Sex variations in the disclosure to parents of same-sex attractions. J. Fam. Psychol..

[51] Hilton A.N., Szymanski D.M. (2011). Family Dynamics and Changes in Sibling of Origin Relationship after Lesbian and Gay Sexual Orientation Disclosure. Contemp. Fam. Ther..

[52] Oswald R. (2000). Family and friendship relationships after young women come out as bisexual or lesbian. J. Homosex..

[53] Legate N., Ryan R.M., Weinstein N. (2012). Is Coming Out Always a “Good Thing”? Exploring the Relations of Autonomy Support, Outness, and Wellness for Lesbian, Gay, and Bisexual Individuals. Soc. Psychol. Personal. Sci..

[54] Grov C., Bimbi D.S., Nanin J.E., Parsons J.T. (2006). Race, ethnicity, gender, and generational factors associated with the coming-out process among lesbian, and bisexual individuals. J. Sex Res..

[55] D’Augelli A.R., Grossman A.H., Starks M.T., Sinclair K.O. (2010). Factors Associated with Parents’ Knowledge of Gay, Lesbian, and Bisexual Youths’ Sexual Orientation. J. GLBT Fam. Stud..

[56] Cain R. (1991). Stigma management and gay identity development. Soc. Work.

[57] Pachankis J.E., Goldfried M.R., Ramrattan M.E. (2008). Extension of the rejection sensitivity construct to the interpersonal functioning of gay men. J. Consult. Clin. Psychol..

[58] Charbonnier É., Graziani P. (2013). Stress, risque suicidaire et annonce de son homosexualité. Serv. Soc..

[59] Pennebaker J. (1993). Putting stress into words: Health, linguistic, and therapeutic implications. Behav. Res. Ther..

[60] Pennebaker J. (1997). Writing about emotional experiences as a therapeutic process. Psychol. Sci..

[61] Ragins B.R. (2004). Sexual orientation in the workplace: The unique work and career experiences of gay, lesbian and bisexual workers. Research in Personnel and Human Resources Management.

[62] Vaughan M. (2007). Coming Out Growth: Conceptualizing and Assessing Experiences of Stress-Related Growth Associated with Coming Out as Lesbian or Gay. Ph.D. Thesis.

[63] Vaughan M.D., Waehler C.A. (2010). Coming Out Growth: Conceptualizing and measuring stress-related growth associated with coming out to others as a sexual minority. J. Adult Dev..

[64] Coursaud J.B. (2002). L’Homosexualité Entre Préjugés et Réalités.

[65] Angst J., Gamma A., Gastpar M., Lepine J.P., Mendlewicz J.T.A. (2002). Gender differences in depression. Eur. Arch. Psychiatry Clin. Neurosci..

[66] Cavanagh J., Carson A., Sharpe M. (2003). Psychological autopsy studies of suicide: A systematic review. Psychol. Med..

[67] Kim C., Lesage A., Seguin M., Chawky N., Vanier C., Lipp O., Turecki G. (2003). Patterns of co-morbidity in male suicide completers. Psychol. Med..

[68] Zhang J., Wieczorek W., Conwell Y., Tu X. (2011). Psychological strains and youth suicide in rural China. Soc. Sci. Med..

[69] Speckens A., Hawton K. (2005). Social problem solving in adolescents with suicidal behavior: A systematic review. Suicide Life-Threat..

[70] Wilson K., Stelzer J., Bergman J. (1995). Problem solving, stress, and coping in adolescent suicide attempts. Suicide Life-Threat. Behav..

[71] Blankstein K., Lumley C., Crawford A. (2007). Perfectionism, hopelessness, and suicide ideation: Revisions to diathesis-stress and specific vulnerability models. J. Ration. Emot. Cognit. Behav. Ther..

[72] Fox R.C. (1995). Bisexual Identities. Lesbian, Gay, and Bisexual Identities over the LifespanPsychological Perspectives.

[73] Fenner Y., Garland S.M., Moore E.E., Jayasinghe Y., Fletcher A., Tabrizi S.N., Gunasekaran B., Wark J. (2012). Web-Based recruiting for Health Research Using a Social Networking Site: An Exploratory Study. J. Med. Internet Res..

[74] Pouchieu C., Castetbon K., Galan P., Hercberg S., Touvier M. (2012). Prise de compléments alimentaires évaluée par autoquestionnaire sur Internet, étude NutriNet-Santé. Rev. Epidemiol. Sante Publique.

[75] LaRose R., Lin C.A., Eastin M.S. (2003). Unregulated Internet Usage: Addiction, Habit, or Deficient Self-Regulation?. Media Psychol..

[76] Mastro D.E., Eastin M.S., Tamborini R. (2002). Internet Search Behaviors and Mood Alterations: A Selective Exposure Approach. Media Psychol..

[77] Byrne S., Morgan S., Fitzpatrick C., Boylan C., Crowley S., Gahan H., Howley J., Staunton D., Guerin S. (2008). Deliberate self-harm in children and adolescents: A qualitative study exploring the needs of parents and carers. Clin. Child Psychol. Psychiatry.

[78] Bull S., McFarlane M., Rietmeijer C. (2001). HIV and sexually transmitted infection risk behaviors among men seeking sex with men on-line. Am. J. Public Health.

[79] Bull S., Lloyd L., Rietmeijer C., McFarlane M. (2004). Recruitment and retention of an online sample for an HIV prevention intervention targeting men who have sex with men: The smart sex quest project. AIDS Care.

[80] Charbonnier E., Graziani P. (2011). La perception de jeunes lesbiennes et gais concernant l’attitude de leurs parents à l’égard de leur homosexualité. Can. J. Community Ment. Health.

